# Sitravatinib, a Tyrosine Kinase Inhibitor, Inhibits the Transport Function of ABCG2 and Restores Sensitivity to Chemotherapy-Resistant Cancer Cells *in vitro*

**DOI:** 10.3389/fonc.2020.00700

**Published:** 2020-05-12

**Authors:** Yuqi Yang, Ning Ji, Qiu-Xu Teng, Chao-Yun Cai, Jing-Quan Wang, Zhuo-Xun Wu, Zi-Ning Lei, Sabrina Lusvarghi, Suresh V. Ambudkar, Zhe-Sheng Chen

**Affiliations:** ^1^Department of Pharmaceutical Sciences, College of Pharmacy and Health Sciences, St. John's University, Queens, NY, United States; ^2^State Key Laboratory of Experimental Hematology Institute of Hematology and Hospital of Blood Diseases, Chinese Academy of Medical Science and Peking Union Medical College, Tianjin, China; ^3^Laboratory of Cell Biology, Center for Cancer Research, National Cancer Institute, NIH, Bethesda, MD, United States

**Keywords:** sitravatinib, tyrosine kinase inhibitor, multidrug resistance, ATP-binding cassette (ABC) transporters, ATP-binding cassette super-family G member 2 (ABCG2)

## Abstract

Sitravatinib, also called MGCD516 or MG-516, is a broad-spectrum tyrosine kinase inhibitor (TKI) under phase III clinical evaluation. Herein, we explored the activity of sitravatinib toward multidrug resistance (MDR) by emphasizing its inhibitory effect on ATP-binding cassette super-family G member 2 (ABCG2). ABCG2 is a member of ATP-binding cassette (ABC) transporter family and plays a critical role in mediating MDR. Sitravatinb received an outstanding docking score for binding to the human ABCG2 model (PDB code: 6ETI) among thirty screened TKIs. Also, an MTT assay indicated that sitravatinib at 3 μM had the ability to restore the antineoplastic effect of various ABCG2 substrates in both drug-selected and gene-transfected ABCG2-overexpressing cell lines. In further tritium-labeled mitoxantrone transportation study, sitravatinib at 3 μM blocked the efflux function mediated by ABCG2 and as a result, increased the intracellular concentration of anticancer drugs. Interestingly, sitravatinib at 3 μM altered neither protein expression nor subcellular localization of ABCG2. An ATPase assay demonstrated that ATPase activity of ABCG2 was inhibited in a concentration-dependent manner with sitravatinib; thus, the energy source to pump out compounds was interfered. Collectively, the results of this study open new avenues for sitravatinib working as an ABCG2 inhibitor which restores the antineoplastic activity of anticancer drugs known to be ABCG2 substrates.

## Introduction

Evidence from clinical studies showed that patients with multidrug resistant (MDR) tumors have a poorer prognosis and decreased likelihood of survival compared to cancer patients with drug sensitive tumors (1). Cancer patients develop cross-resistance to various structurally and functionally unrelated chemotherapeutic agents, resulting in treatment failure (2, 3). Overexpression of ATP-binding cassette (ABC) transporters is a leading cause of MDR (4). ABC transporters can be divided into seven subfamilies (ABCA to ABCG). The overexpression of certain transporters leads to MDR, including, but not limited to, ABCB1 (P-glycoprotein, P-gp), ABCG2 (breast cancer resistance protein, BCRP/MXR) and ABCC1 (multidrug resistance-associated protein 1, MRP1) (4, 5). Functionally, ABCB1 and ABCG2 work as efflux pumps, and are located in the lipid raft of specific cell lines (1, 6), reducing the intracellular level of various antineoplastic agents accumulating in cancer cells.

Currently, there are several approaches to circumvent MDR and to enhance the efficacy of antineoplastic drugs such as chemosensitizers (7), gene therapy (8, 9), immune-oncology (10), nanotechnology (11), or traditional Chinese medicine (12). The chemosensitizers, fumitremorgin C (FTC) and its derivative Ko143 are commonly used reference inhibitors of the ABCG2 transporter (13). However, FTC has neurotoxic effects and was withdrawn from clinical use (13). Furthermore, Ko143 was shown to lack specificity for the ABCG2 transporter at high concentrations and it was metabolically unstable in plasma (14). Therefore, more specific and less toxic inhibitors of ABC transporters are urgently needed for both preclinical and clinical evaluation.

*In vitro* studies have shown that some, but not all, novel tyrosine kinase inhibitors (TKIs) have ability to inhibit the ABCG2 transporter (15, 16). Clinically, TKIs are used as first- or second- line treatments for certain metastatic cancers (16, 17). However, TKIs have non-specific and “off-target” effects (18), thereby probably explaining why TKIs [1] are used as alternative treatments in the clinical setting and [2] restore the anticancer efficacy of chemotherapeutic drugs in the ABCG2-mediated MDR model.

Sitravatinib, also called MGCD516 or MG-516, is a broad-spectrum TKI targeting MET, TAM (TYRO3, AXL, MerTK), and members of vascular endothelial growth factor receptor (VEGFR), platelet-derived growth factor receptor (PDGFR), and Eph families (17, 19, 20). Notably, it has been reported that sitravatinib has potent antitumor efficacy, that may be due, in part, to altering the tumor microenvironment and restoring the efficacy of immune checkpoint blockade (PD-1) in diverse cancer models (20). Dolan et al. reported that sitravatinib could combat drug resistance caused by sunitinib and axitinib in metastatic human and mouse models (17). Together, all these studies provide us with a clue that sitravatinib has the capability to antagonize MDR in cancer cells. Thus, various studies indicate that sitravatinib is efficacious in reversing or antagonizing MDR in cancer cells. Furthermore, sitravatinib is under nine ongoing clinical trials for various indications, with one being a phase III study (NCT03906071). To date, these studies have proved that intolerable adverse effects or unacceptable toxicity profile are not found under sitravatinib treatment in preclinical or clinical model. In this article, we focus on the antagonizing activity of sitravatinib toward MDR mediated by ABCG2.

## Materials and Methods

### Chemicals and Reagents

Sitravatinib was purchased from ChemieTek (Indianapolis, IN). Gilteritinib, BMS-777607, merestinib, and LOXO-101 were kindly provided as free samples from Selleckchem (Houston, TX). Topotecan was purchased from Selleckchem (Houstin, TX). Fetal bovine serum (FBS) was purchased from Atlanta Biologicals (Atlanta, GA). Dulbecco's modified Eagle medium (DMEM), antibiotics (penicillin/streptomycin [P/S]), and trypsin were obtained from Corning (Corning, NY). Mitoxantrone and SN-38 were purchased from Medkoo Sciences (Chapel Hill, NC). Phosphate buffered saline (PBS) (pH 7.4) was obtained from VWR Chemicals (Solon, OH). Ko143, cisplatin, and G418 were obtained from Enzo Life Sciences (Farmingdale, NY). Dimethyl sulfoxide (DMSO), 3-(4,5-dimethylthiazol-yl)-2,5-diphenyltetrazolium bromide (MTT) and Triton X-100 were purchased from Sigma-Aldrich (St. Louis, MO). Formaldehyde was obtained from J.T. Baker Chemical (Phillipsburg, NJ). Bovine serum albumin (BSA), 4′,6-diamidino-2-phenylindole (DAPI), PageRuler^TM^ plus pre-stained protein ladder, GAPDH loading control monoclonal antibody (GA1R), Pierce^TM^ ECL Western blotting substrate, Alexa Fluor 488 conjugated goat anti-mouse IgG secondary antibody, and liquid scintillation cocktail were purchased from Thermo Fisher Scientific (Rockford, IL). HRP-conjugated rabbit anti-mouse IgG secondary antibody was purchased from Cell Signaling Technology (Dancers, MA). The monoclonal anti-BCRP antibody (BXP-21) was obtained from Millipore (Billerica, MA). [^3^H]-Mitoxantrone (0.5 Ci·mmol^−1^) were purchased from Moravek Biochemicals (Brea, CA).

### Cell Lines and Cell Culture

The non-small cell lung cancer (NSCLC) cell line, NCI-H460, and the corresponding mitoxantrone-selected NCI-H460/MX20 cells were used. The NCI-H460/MX20 cells were developed and maintained in complete medium containing 20 nM of mitoxantrone and these cells were shown to overexpress the wild-type ABCG2 protein (21). The human colon carcinoma cell line, S1, and its corresponding mitoxantrone-selected S1-M1-80 cells were used. The S1-M1-80 cells were selected and maintained in complete medium containing 80 μM of mitoxantrone and were shown to overexpress a mutant allele (R482G) in the ABCG2 gene (22, 23). In addition, transfected cells were also used in this article. HEK293/pcDNA3.1, HEK293/ABCG2-482-R2, HEK293/ABCG2-482-G2, and HEK293/ABCG2-482-T7 were transfected with either an empty vector pcDNA3.1 or a pcDNA3.1 vector containing a full length ABCG2 encoding arginine (R), glycine (G), or threonine (T) for amino acid at position 482 (24). All transfected cell lines were selected and cultured in complete medium with 2 mg·ml^−1^ of G418. All cell lines were cultured in DMEM complete medium containing 10% FBS and 1% P/S at 37°C in a humidified incubator supplied with 5% CO_2_. All drug-resistant cells were grown in drug-free medium for more than 3 weeks and passaged at least 3 generations before experimental use. All drug-selected and gene-transfected cell lines used in this article were kindly provided by Drs. Susan Bates and Robert Robey (NCI, NIH, Bethesda, MD).

### Induced-Fit Docking Analysis and Molecular Dynamic Simulations for ABCG2

Previously reported protocol was used for the molecular docking simulations (16) by using Maestro v11.1 software (Schrödinger, LLC, New York, NY). The structure of sitravatinib after preparation by LigPrep v4.1 to simulate the low-energy pose was subjected to the Glide XP (extra precision) docking default protocol (Schrödinger, LLC, New York, NY) with a pre-prepared cryo-EM structure of the human ABCG2 model (PDB code: 6ETI). The human ABCG2 protein model was confined to the docking grid at the drug-binding cavity by selecting specific amino acids that were deemed to be involved in specific interactions (25). The following induced-fit docking (IFD) was generated based on the best scored results from the Glide XP analysis. The docked sitravatinib-ABCG2 complex simulated based on IFD was then subjected to another 10 ns molecular dynamic (MD) simulation using a previously reported default protocol (26). The docking scores were calculated and reported as kcal·mol^−1^ and the highest-scoring result was used for further graphical analysis.

### Cell Viability Assay

A modified MTT colorimetric assay was conducted to determine the cytotoxic efficacy of the chemotherapeutic agents with or without ABCG2 inhibitors, as described previously (26). In short, each cell line was harvested and seeded evenly into a 96-well plate with a density of 5 × 10^3^ cell·well^−1^. The next day, cells were pretreated for 2 h with or without sitravatinib, or Ko143, at the indicated concentrations. Following pretreatment, cells were co-cultured with anticancer drugs (mitoxantrone, SN-38, topotecan or cisplatin) at serial concentrations. After a 68 h incubation period, an MTT solution (4 mg·ml^−1^ in PBS) was added to each well and the cells were further incubated at 37°C for 4 h in the dark. The supernatant was removed, and the resulting formazan crystals were dissolved with DMSO. An accuSkan^TM^ GO UV/Vis Microplate Spectrophotometer (Fisher Sci., Fair Lawn, NJ) was used to measure the absorbance at 570 nm. The half maximal inhibitory concentration (IC_50_) for each anticancer drug was calculated as previously described (27). DMSO was used as an effective solvent to prepare stock solution (stock concentration is 10 mM) of all compounds. As the highest final concentration in cell viability assay was 100 μM, the final concentration of solvent was 1% in treatment medium (DMEM complete medium containing 10% FBS and 1% P/S). Notably, the control group was treated with solvent only. All experiments were performed independently at least three times performed in triplicate.

### [^3^H]-Mitoxantrone Accumulation and Efflux Assay

The detailed protocol as previously stated was followed (28). Briefly, ABCG2-mediated MDR cells (1 × 10^6^ cell·well^−1^) were seeded into a 24-well plate and incubated 1 day prior to further study. After 2 h of incubation in a drug-free medium, sitravatinib (0.75 and 3 μM) or a known inhibitor (3 μM), cells were co-incubated with [^3^H]-mitoxantrone at 37°C for another 2 h. The cells were then washed twice with ice-cold PBS, trypsinized, harvested, and placed in 5 ml of a liquid scintillation cocktail. Radioactivity was measured using a Packard TRI-CARB 1,900 CA liquid scintillation analyzer (Packard Instrument, Downers Grove, IL).

To determine the effect of sitravatinib on the efflux function of the ABCG2 transporter, the tritium-labeled mitoxantrone efflux assay was performed as previously stated (29). As described in the accumulation phase above, cells were pretreated with or without an ABCG2 inhibitor for 2 h followed by 2 h co-treatment with [^3^H]-mitoxantrone. The cells were then incubated in the presence or absence of an inhibitor at serial time points (0, 30, 60, and 120 min). Subsequently, the cells were washed twice with iced PBS, trypsinized, and transferred into a 5 ml liquid scintillation cocktail. Finally, radioactivity was measured as described above. In this assay, Ko143 served as the reference ABCG2 inhibitor for MDR cell lines. All the experiments were conducted independently three times.

### Immunoblotting Analysis

Immunoblotting analysis (i.e., Western blotting) was conducted to detect the protein expression level of ABCG2 after up to 72 h of incubation with or without sitravatinib at 3 μM using a previously reported protocol (26). Briefly, following incubation with the highest non-toxic concentration of sitravatinib for 0, 24, 48, or 72 h, cells were harvested and lysed with lysis buffer (10 mM Tris, 1 mM EDTA, 0.1% SDS, 150 mM NaCl, 1% Triton X-100 and protease inhibitor cocktail) on ice for 20 min, followed by centrifugation at 4°C at 15,000 G for 20 min. The supernatant was collected and a bicinchoninic acid (BCA) Protein Kit (Thermo Scientific, Waltham, MA) was used to quantify the total concentration of protein in each sample. Equal amounts of total protein (20 μg) were loaded and separated by sodium dodecyl sulfate polyacrylamide gel electrophoresis (SDS-PAGE), followed by transfer onto a polyvinylidene fluoride (PVDF) membrane (Millipore, Billerica, MA). After blocking with 4% non-fat milk for 2 h at room temperature, the membrane was incubated with primary monoclonal antibodies against ABCG2 (at 1:500 dilution) and GAPDH (at 1:2,000 dilution) at 4°C overnight. The next day, after washing three times with Tris buffered saline containing 0.4% Tween 20 (TBST), the membrane was incubated with an HRP-conjugated secondary antibody (at 1:2,000 dilution) for 2 h at room temperature. Finally, the chemiluminescence signal of protein-antibody complex was developed by ECL substrate as per manufacturer's instruction. The relative density of each protein band was analyzed by ImageJ software (NIH, Bethesda, MD). All experiments were repeated three times independently.

### Immunofluorescence Assay

Immunofluorescence assay was performed to assess the subcellular localization of membrane protein ABCG2 as previously described (30). In short, each cell line (1 × 10^4^ cell·well^−1^) was seeded onto a 24-well plate and treated with or without sitravatinib at 3 μM for several time frames (0, 24, 48, and 72 h). After the treatment period, cells were washed with PBS three times, fixed in 4% paraformaldehyde for 15 min, and permeabilized by 0.1% Triton X-100 for 15 min before being blocked with 6% BSA for 2 h. Subsequently, the presence of ABCG2 was detected using anti-BCRP monoclonal antibody (at 1:1,000 dilution) followed by Alexa Fluor 488 conjugated secondary antibody (at 1:1,000 dilution). Finally, 1 μg·ml^−1^ DAPI was used to counterstain the nuclei. The immunofluorescence images were captured via an EVOS FL Auto fluorescence microscope (Thermo Scientific, Waltham, MA). All the experiments were performed three times independently.

### ATPase Assay

The ATPase activity was determined by quantifying the amount of product (inorganic phosphate, P_i_) produced after ATP hydrolysis. High Five insect cells expressing ABCG2 were used to prepare membrane vesicles (4). Next, membrane vesicles (10 μg total protein) were combined with assay buffer containing 50 mM MES (pH 6.8), 50 mM KCl, 5 mM sodium azide, 2 mM EGTA, 2 mM DTT, 1 mM ouabain, and 10 mM MgCl_2_, final volume 100 μL. Then sitravatinib was incubated with the membrane vesicles for 3 min at 37°C. The ATP hydrolysis was initiated by addition of 5 mM of Mg-ATP. After incubation at 37°C for 20 min, the reaction was terminated by addition of 100 μl 5% SDS solution. The amount of P_i_ was quantified using the method modified from Murphy and Riley (31), which is based on the complex formation of phosphate with potassium-antimonyl-tartarate in acidic ammonium molybdate. After reduction induced by ascorbic acid, the complex displays a stable blue color that was quantified by measuring the absorbance at 880 nm using a spectrophotometer (Bio-Rad, Hercules, CA).

### Data and Statistical Analysis

All data are presented as the mean ± SD (standard deviation) obtained from three independent experiments performed in triplicate. Comparisons were made between the control group and the corresponding treatment group. The results were analyzed with one-way or two-way analysis of variance (ANOVA) following Tukey *post hoc* analysis. The statistical analysis was performed by GraphPad Prism version 6.02 for Windows (GraphPad Software, La Jolla, CA). The *a priori* significance level was *p* < 0.05.

## Results

### Pre-selecting TKIs Using a Molecular Simulation Analysis and a Modified Cell Viability Assay

**Figure 1:**
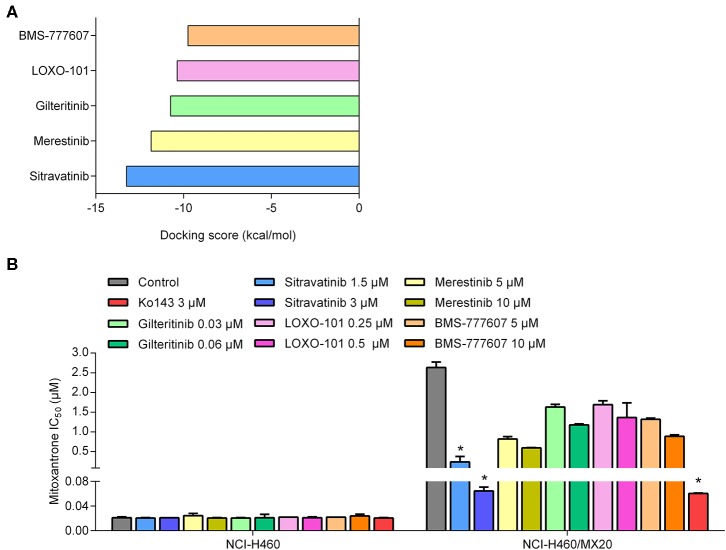
The preliminary study to pre-select possible ABCG2 inhibitor. **(A)** The docking scores of TKIs were screened by computational simulation analysis. **(B)** The antagonizing activity of TKIs toward MDR cell lines mediated by ABCG2. Data are represented as mean ± SD from at least three independent assays. *p < 0.05 compared with control group.

To predict and compare the possible interaction between thirty TKIs and the human ABCG2 model, an *in silico* screening was conducted. The glide gscores of all screened TKIs are shown in Table S1. Based on the results from a preliminary study, sitravatinib had a higher docking score for binding to ABCG2 (Figure 1A) and a greater efficacy in reversing the anticancer efficacy of mitoxantrone in ABCG2-overexpressing cell line compared to other TKIs (BMS-777607, LOXO-101, gilteritnib, and merestinib) (Figure 1B). Based on these results, we conducted experiments to determine if sitravatinib was efficacious in attenuating ABCG2-mediated drug resistance in cell lines overexpressing the ABCG2 transporter.

### Molecular Docking Sitravatinib in the Drug-Binding Pocket of ABCG2

**Figure 2:**
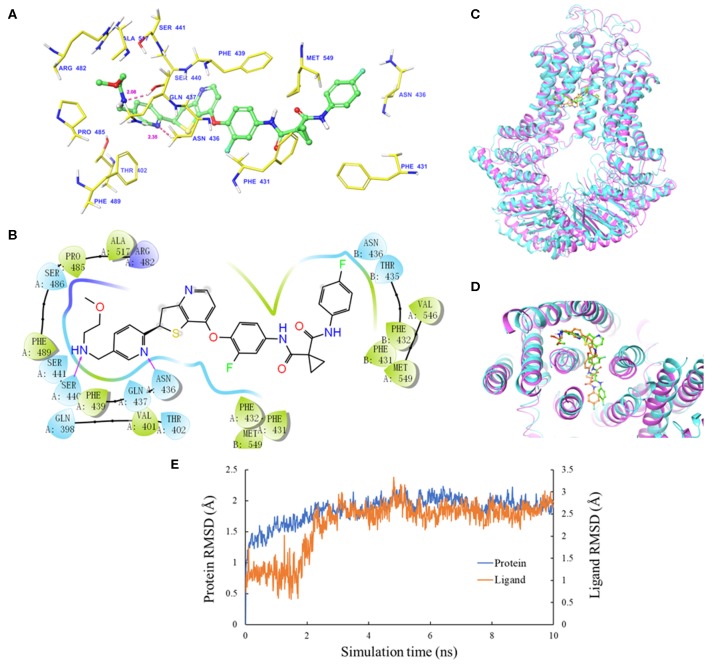
Molecular docking analysis with sitravatinib. **(A)** The docked conformation of sitravatinib (ball-and-stick model) is shown within the ABCG2 drug-binding cavity, with the atoms colored as follows: carbon, green; hydrogen, white; oxygen, red; nitrogen, blue. Important amino acid residues are described with the same color scheme as above for all atoms, except for carbon atoms in yellow. Dotted pink lines represent hydrogen-bonding interactions and the values of the correlation distances are indicated in Å. **(B)** The 2D schematic diagram of ligand–receptor interaction between sitravatinib and the human ABCG2 model. Amino acids within 3 Å are indicated as colored bubbles, polar residues are light blue, hydrophobic residues are green, and the positive charged residue is dark blue. Purple arrows denote H-bonds. **(C,D)** The superimposition of MD pose of sitravatinib within the binding cavity of ABCG2. Sitravatinib molecules are depicted as ball and stick model in faded green or orange for pre- and post-MD, respectively. The ABCG2 structure is in ribbon diagram in faded magenta or faded cyan for pre- and post-MD, respectively. **(E)** RMSD trajectory of ABCG2 and sitravatinib in sitravatinib-ABCG2 complex over the 10 ns simulation run.

The IFD was carried out to simulate the interactions between sitravatinib and the atomic structure of human ABCG2 homodimer (chain A and B). As shown in Figure 2A, the lowest energy binding pose of sitravatinib was predicted in the drug-binding cavity through hydrophobic interactions with nearby amino acids. Two hydrogen bonds were formed between the ethylamino group and SER440 of ABCG2 chain A, and between the nitrogen in the pyridine ring of sitravatinib and ASN436 of ABCG2 chain A, respectively. Sitravatinib produced the best glide gscore out of all TKIs with a gscore of −13.248 kcal·mol^−1^, which is indicative of the free binding energy of the ligand. Figure 2B shows that the best-scored pose of sitravatinib occurs in the ABCG2 transmembrane domain with residues GLN 398, VAL 401, THR 402, PHE431, PHE 432, ASN 436, GLN 437, PHE 439, SER 440, SER 441, ARG 482, SER 486, PRO 485, PHE 489, ALA 517, VAL 546, and MET 549 in protein chain A, and PHE 431, PHE 432, THR 435, ASN 436, and MET 549 in protein chain B. Further molecular dynamic simulations of the sitravatinib-ABCG2 complex, as shown in Figures 2C,D, indicated that the position of sitravatinib in binding pocket did not show significant movements or changes after 10 ns. By analyzing the root mean square deviation (RMSD) of protein-drug complex, both the backbone of the protein and the complex were shown to be in a stable conformation after the first 2 ns and maintained this conformation until the end of the simulation (Figure 2E).

### Effect of Sitravatinib on the Efficacy of Antineoplastic Drugs in ABCG2-Mediated MDR Cell Lines

**Figure 3:**
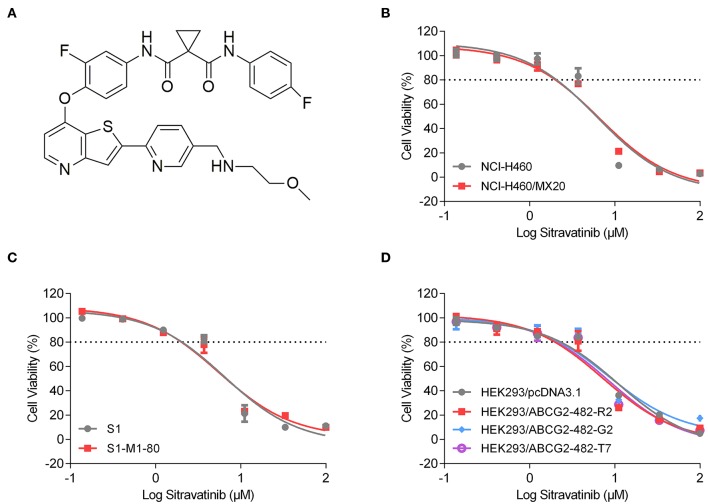
Chemical structure and the cytotoxic activity of sitravatinib in ABCG2-overexpressing cell lines and their corresponding sensitive cell lines. **(A)** Chemical structure of sitravatinib. The cell viability-concentration curves for NCI-H460/MX20 and NCI-H460 **(B)**, S1-M1-80 and S1 **(C)**, HEK293 cell lines transfected with full length ABCG2 or empty vector **(D)** after treatment with serial concentrations of sitravatinib for 72 h. Data are shown as mean ± SD, obtained from at least three experiments performed independently.

An MTT assay was used to obtain concentration-dependent cell viability curves (concentration range for sitravatinib from 0 to 100 μM) and the non-toxic concentrations for further reversal study. Based on cytotoxicity results, 3 μM concentration was chosen as the highest non-toxic concentration of sitravatinib for further reversal studies (chemical structure of the compound is shown in Figure 3A). At this concentration, the cell survival rate was more than 80% after 72 h treatment period (indicated as dash line in Figures 3B–D).

**Figure 4:**
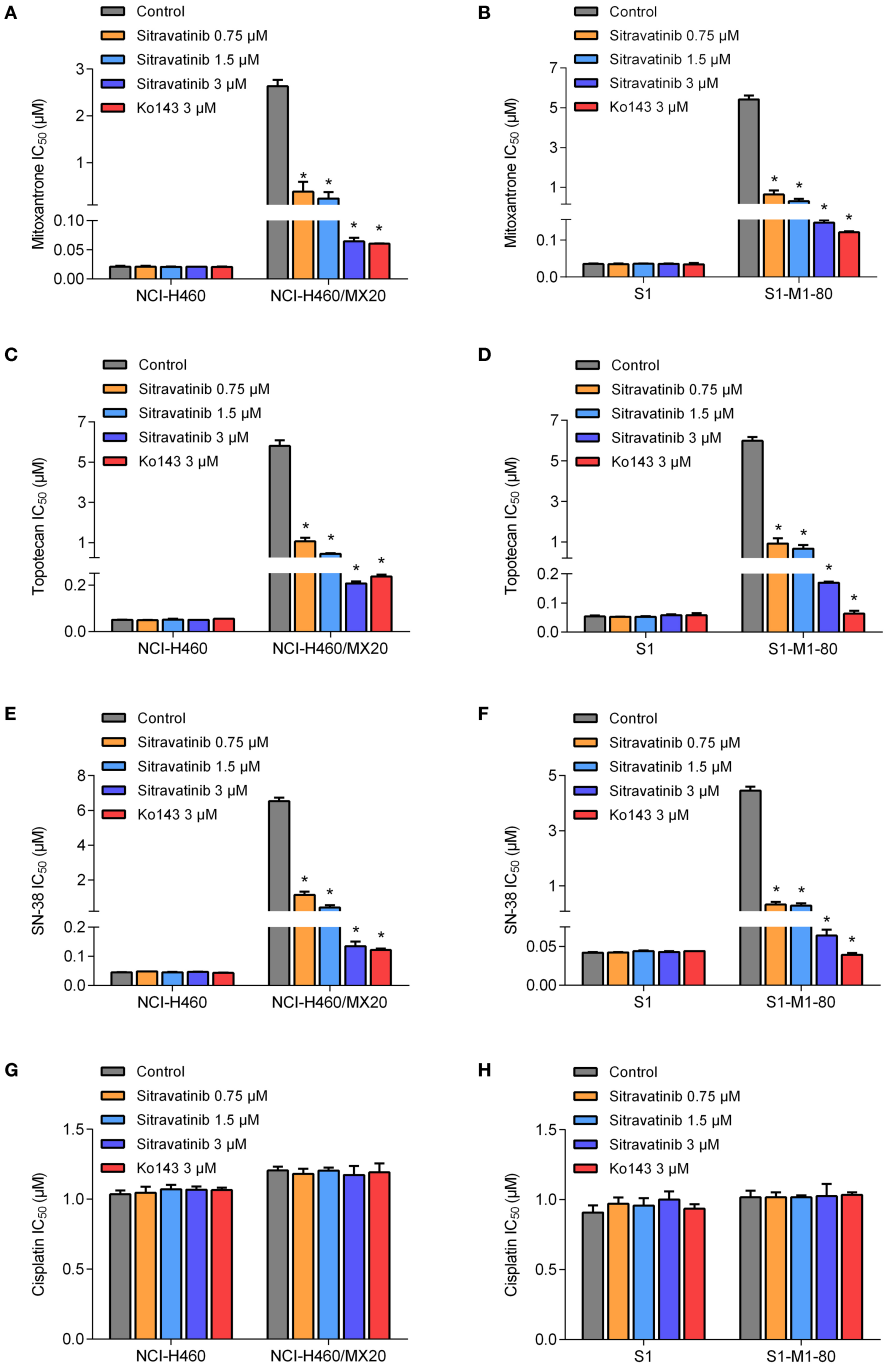
Drug sensitivity of drug-selected ABCG2-overexpressing cell lines to various chemotherapeutic agents in the absence or presence of sitravatinib. The IC_50_ values of mitoxantrone **(A,B)**, topotecan **(C,D)**, SN-38 **(E,F)**, and cisplatin **(G,H)** in NCI-H460 and its mitoxantrone-selected resistance cell line (NCI-H460/MX20), S1 and its mitoxantrone-selected resistance cell line (S1-M1-80). Data were collected from independent experiments repeated at least three times and are shown as mean ± SD. *p < 0.05 compared with control group.

**Figure 5:**
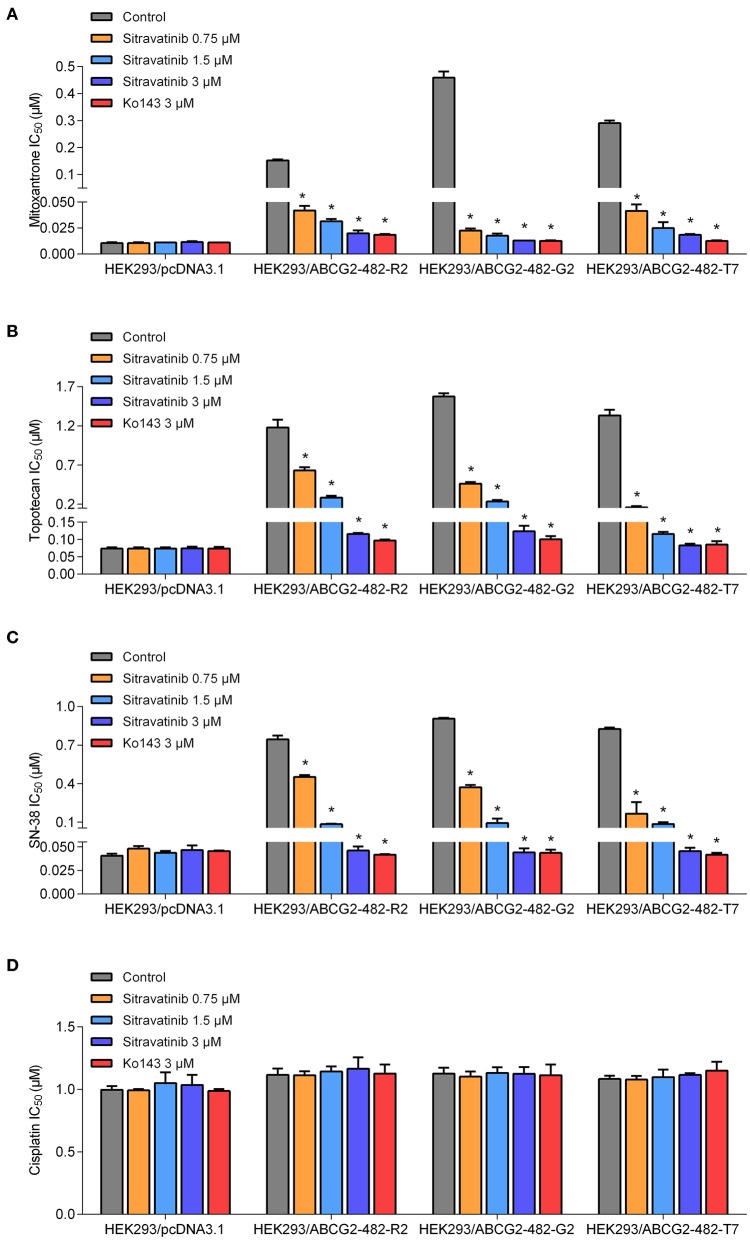
The antagonizing effect of sitravatinib in ABCG2-transfected HEK293 cell lines and the parental cell line HEK293/pcDNA3.1. The IC_50_ values of mitoxantrone **(A)**, topotecan **(B)**, SN-38 **(C)**, and cisplatin **(D)** in transfected cell lines. Data are presented as mean ± SD. All data were obtained from at least three independent assays. *p < 0.05 compared with control group.

The reversal study showed that sitravatinib made the NCI-H460/MX20 and S1-M1-80 cells became more sensitive to mitoxantrone, SN-38 and topotecan in a concentration-dependent manner (Figures 4A–F). Additionally, it increased the efficacy of these antineoplastic agents in ACBG2-transfected HEK293 cell lines, including HEK293/ABCG2-482-R2, HEK293/ABCG2-482-G2, and HEK293/ABCG2-482-T7 (Figures 5A–C). Importantly, sitravatinib had a significant reversal effect on both wild-type and mutant ABCG2 overexpressing cells, whereas this effect could not be found in corresponding parental cells NCI-H460, S1, or HEK293/pcDNA3.1. It is notable that the reversal activity in ABCG2-overexpressing cell lines is comparable to its counterpart positive control treatment Ko143 at the same concentration level, which served as a reference inhibitor of ABCG2. In addition, sitravatinib did not affect the anticancer effect of cisplatin, a drug that is not the substrate of ABCG2, in neither drug-sensitive nor ABCG2-overexpressing cell lines (Figures 4G,H, 5D). The fold-reduction of IC_50_ values for chemotherapeutic drugs with or without inhibitors are shown in Tables S2, S3.

Overall, these results denoted that sitravatinib can improve the efficacy of multiple chemotherapeutic agents in ABCG2-mediated MDR cell lines in a concentration-dependent manner.

### Modulation of ABCG2-Mediated Efflux of [^3^H]-Mitoxantrone in MDR Cell Lines

In order to understand the mechanism of action of sitravatinib in reversing MDR, several mechanism-based assays were performed. To evaluate the function of ABCG2, the intracellular concentration of tritium-labeled chemotherapeutic drug was quantified in parental cells and cells overexpressing ABCG2 with or without an inhibitor.

**Figure 6:**
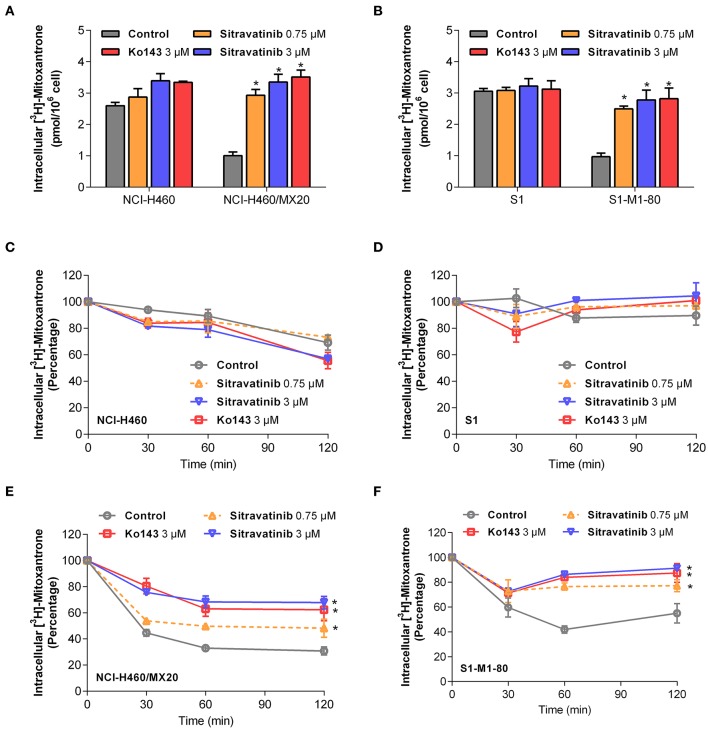
Sitravatinib inhibits ABCG2-mediated mitoxantrone transport. The accumulation of tritium-labeled mitoxantrone in NCI-H460 and NCI-H460/MX20 **(A)**, S1 and S1-M1-80 **(B)**. The ABCG2-mediated efflux activity in NCI-H460 **(C)**, S1 **(E)** and their corresponding mitoxantrone-selected cell line NCI-H460/MX20 **(D)**, S1-M1-80 **(F)** at a series of time points (0, 30, 60, 120 min). Data from at least three independent experiments are shown as mean ± SD. *p < 0.05 compared with control group.

Sitravatinib at 3 μM concentration significantly increased the intracellular [^3^H]-mitoxantrone accumulation level from 38.7 to 129.2% or from 31.6 to 91.0% in NCI-H460/MX20 or S1-M1-80 cells, respectively, but not in the corresponding parental cell line counterparts (NCI-H460 and S1), as shown in Figures 6A,B. In this study, Ko143 served as reference ABCG2 inhibitor. Hence, these results suggested that sitravatinib increases the intracellular accumulation of [^3^H]-mitoxantrone in ABCG2-mediated MDR cells.

Furthermore, in order to further understand the enhanced intracellular accumulation of [^3^H]-mitoxantrone in MDR cells, a time-course study was conducted to assess the efflux function of ABCG2 by quantifying the level of intercellular [^3^H]-substrate at serial time points in the presence or absence of inhibitors. As shown in Figures 6E,F, after 120 min, NCI-H460/MX20 and S1-M1-80 cells without ABCG2 inhibitor treatment maintained only 30 and 55% of [^3^H]-mitoxantrone, respectively. On the contrary, when treated with 3 μM sitravatinib, 68 and 91% [^3^H]-mitoxantrone was accumulated in NCI-H460/MX20 and S1-M1-80 cells, respectively. In contrast, sitravatinib did not significantly change the efflux function in parental cells (NCI-H460 or S1) at different time points (Figures 6C,D).

Taken together, these results demonstrated that sitravatinib could enhance the intracellular accumulation of a tritium-labeled chemotherapeutic drug by blocking the efflux function mediated by ABCG2.

### Effect of Sitravatinib on Expression and Localization of ABCG2 in ABCG2-Mediated MDR Cell Lines

It is known that the reversal mechanism of action could involve either downregulation of protein expression level and/or alternation of subcellular localization of the transporter (4). Therefore, immunoblotting analysis and immunofluorescence assay were conducted to detect the expression and localization of ABCG2 protein, respectively.

**Figure 7:**
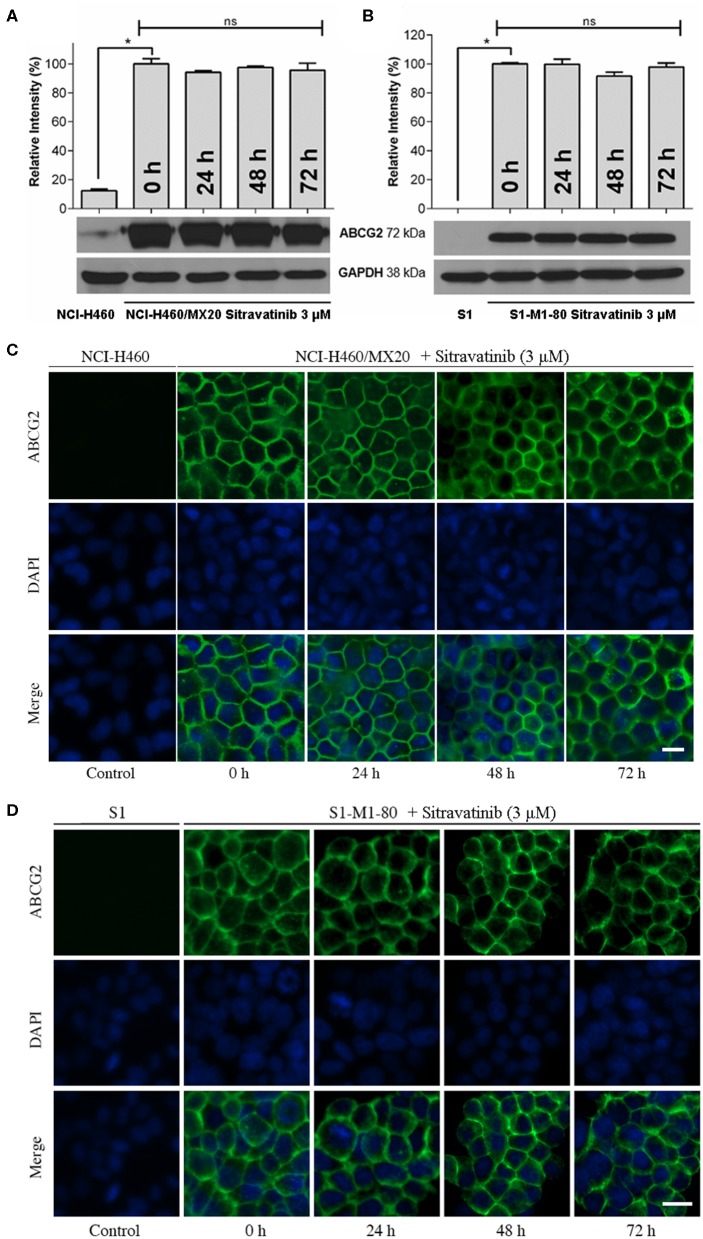
Sitravatinib does not affect protein expression level nor the subcellular localization of ABCG2. Immunoblotting analysis showing the expression level of ABCG2 in MDR cell lines overexpressing ABCG2 NCI-H460/MX20 **(A)** and S1-M1-80 **(B)** compared to the corresponding parental cell lines. The relative density of each band is shown as mean ± SD, collected from at least three independent assays. *p < 0.05 compared with control group. Immunofluorescence assay indicating the subcellular localization of membrane protein ABCG2 of NCI-H460/MX20 **(C)** and S1-M1-80 **(D)** and the corresponding parental cell lines. Color: ABCG2 (Green), DAPI (Blue). Scale bar:10 μm.

The expression level of ABCG2 in NCI-H460/MX20 and S1-M1-80 was not significantly altered even after 72 h treatment with 3 μM sitravatinib, Figures 7A,B. In addition, ABCG2 was detected on the cell membrane surface after the cells treated with 3 μM sitravatinib for 0, 24, 48, or 72 h, Figures 7C,D.

Therefore, long-term treatment at the highest non-toxic concentration of sitravatinib (3 μM) neither downregulates the expression level nor affects the subcellular localization of ABCG2 in ABCG2-mediated MDR cell lines.

### Effect of Sitravatinib on ABCG2 ATPase Activity

**Figure 8:**
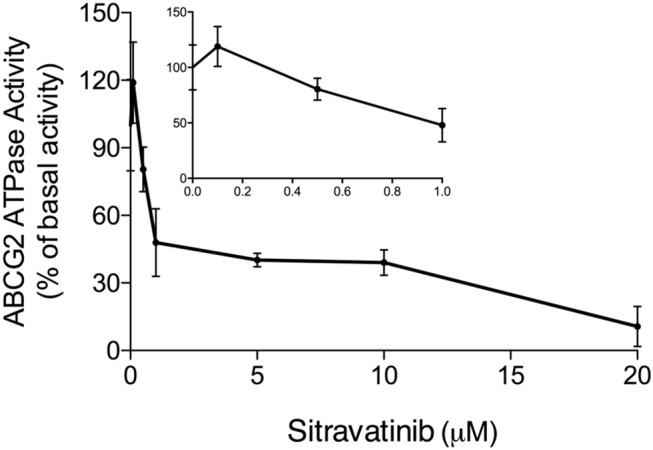
Sitravatinib inhibits ABCG2 ATPase activity in a concentration-dependent manner. The effect of sitravatinib on ATPase activity of ABCG2 in insect cell membrane vesicles was determined as described in the Materials and Methods Section. Data were obtained from independent assays repeated at least three times and are presented as mean ± SD.

It is documented that ATP hydrolysis is the energy source for ABC transporters to pump out endogenous and exogenous toxicants (32, 33). Hence, the effect of sitravatinib on ABCG2 ATPase activity was evaluated. Herein, the ABCG2-mediated ATP hydrolysis was measured in membrane vesicles after incubation with serial concentrations of sitravatinib (0–20 μM). Sitravatinib inhibited 56.2% of the basal ATPase activity, and the inhibitory effect reached 50% at 0.9 μM, see Figure 8. These results indicate that sitravatinib inhibits ABCG2 ATPase activity in a concentration-dependent manner. ATPase data combined with molecular docking suggest that sitravatinib interacts with the drug-binding pocket of the ABCG2.

## Discussion and Conclusion

It is well-known that ABC transporters contribute to MDR and as a result, limit the anticancer efficacy of numerous chemotherapeutic agents in the clinical setting. Due to their efflux function, ABC transporters can extrude many structurally and functionally unrelated anticancer drugs (34), resulting in poor prognosis and low survival rates in cancer patients. In the past decades, many researchers have attempted to synthesize or screen potential inhibitors of ABC transporters to reverse MDR (16, 35–38). However, high toxicity and drug-drug interactions remain to be a challenge (39). To date, many researchers using *in vivo* and *ex vivo* models have demonstrated that TKIs have ability to restore the sensitivity of substrate antineoplastic drugs of ABC transporters for effective chemotherapy (40–43). Most recently, Chen et al. conducted a phase I clinical evaluation in a population of patients to reveal that cyclosporine A (CsA), a competitive ABCB1 inhibitor, could combat drug resistance caused by brentuximab vedotin in relapsed/refractory Hodgkin lymphoma with tolerable and feasible profile (44). These studies provided growing evidence that inhibitors of ABC transporters have promising possibility in the future preclinical and clinical use. Thus, it is meaningful to find potential inhibitors of ABC transporters even though many obstacles exist. It has been documented that some TKIs could behave as substrates or inhibitors of ABC transporters depending on different settings (45). Collectively, this suggests that TKIs with inhibitory activity toward ABCG2 in combination with conventional chemotherapeutic agents can be a promising strategy to circumvent MDR.

Using molecular docking and cell viability assay we first screened thirty TKIs that share active pharmacophoric features of ABC transporter inhibitors, such as the aromatic system, benzamido groups, or methoxyphenyl groups (46). The five compounds with the highest glide gscores were chosen to further examine their MDR reversal activity in cell lines expressing ABCG2. These screening assays showed that sitravatinib had an outstanding docking score within the drug-binding pocket in the transmembrane domain of the homodimer of human ABCG2, and also demonstrated excellent inhibitory activity in the ABCG2-mediated MDR cell lines.

*In silico* analysis of the simulated molecular docking showed specific interactions between sitravatinib and the human ABCG2 drug-binding pocket. The high glide gscore (−13.248 kcal·mol^−1^) suggested strong affinity of sitravatinib. The best-scored pose of other known ABCG2 inhibitors, such as selonsertib, ulixertinib and NVP-TAE684, received glide gscores of −12.278, −11.501, and −12.929 kcal·mol^−1^, respectively (4, 28, 47), which indicates that the affinity of sitravatinib and ABCG2 protein model may be comparable to other known ABCG2 inhibitors. To verify whether this docking ligand could be removed from the binding pocket, further MD simulations were performed. The MD simulations indicated that the binding complex did not change significantly in 10 ns, with the maximal RMSD of sitravatinib being approximately 2 Å. These results suggested that sitravatinib could bind stably to the substrate-binding cavity of human ABCG2 with high affinity.

Cell viability assay demonstrated that the highest non-toxic concentration of sitravatinib was 3 μM. Furthermore, the modified MTT colorimetric assay on ABCG2-mediated MDR cell lines supported the conclusion that the inhibitory effect of sitravatinib was strictly associated with the expression of ABCG2. This was further corroborated using ABCG2-transfected HEK293 cell lines, in which ABCG2 is the solo contributor to MDR. By contrast, the reversal effect of sitravatinib was not found in any of the sensitive cell lines. Notably, sitravatinib did not change the IC_50_ values of cisplatin, which is not a substrate of ABCG2.

A mechanism-based assay was conducted to examine the underlying mechanism of action of the antagonizing activity of sitravatinib in ABCG2-overexpressing cell lines. ABCG2 was shown as an efflux pump (48). Hence, tritium-labeled mitoxantrone-mediated accumulation and efflux assays were conducted to evaluate the pump function of ABCG2. Pre-treatment of the ABCG2 overexpressing cell lines with sitravatinib significantly improved the intracellular accumulation of mitoxantrone, which is a well-established substrate of ABCG2, but not in the corresponding sensitive cell lines. In addition, the ABCG2-expressing cell lines effluxed less amount of the chemotherapeutic drug after incubation with sitravatinib at non-toxic concentrations, compared with their counterparts in parental cell lines. This demonstrated that sitravatinib could impede the ABCG2 efflux function and in turn increase the intracellular accumulation of anticancer drug; thus, sitravatinib at non-toxic concentrations could remarkably antagonize ABCG2-mediated MDR and improve the antineoplastic efficacy of chemotherapeutic agents.

Mechanistically, it is well-documented that several MDR inhibitors downregulate the level of ABCG2 in the plasma membrane to hinder its pump function (4, 15, 49). To evaluate if this is the case with sitravatinib, Western blotting and immunofluorescence assays were conducted. Our results show that sitravatinib did not alter the expression level or the subcellular localization of ABCG2 in ABCG2-expressing cell lines. Collectively, we summarized that sitravatinib directly inhibits the pump function of ABCG2 without changing the expression level or its subcellular localization. Furthermore, the hydrolysis of ATP is the energy source for ABC transporter-mediated efflux of endogenous and exogenous toxicants (32, 33). Therefore, an ATPase assay was performed to determine the effect of sitravatinib on the ATPase activity of ABCG2. The results demonstrated that sitravatinib could partially inhibit ABCG2 ATPase activity suggesting that sitravatinib has the ability to interfere with the transport function of ABCG2 by interfering with its ATPase activity.

In conclusion, our study revealed that sitravatinib is a potential ABCG2 inhibitor with an acceptable toxicity profile. The sensitizing effect of sitravatinib toward different MDR cell lines is due to inhibition of ABCG2 function. In the future, we will focus on long-term exposure to sitravatinib *in vivo* and *ex vivo* to further evaluate the antagonizing effect and its toxicity profile as an ABCG2 inhibitor.

## Data Availability Statement

The datasets generated for this study are available on request to the corresponding author.

## Author Contributions

YY and Z-SC designed the study. YY, NJ, Q-XT, and J-QW gave contribution to perform experiments. C-YC, Z-NL, Z-XW, SL, and SA provided technical and material support. YY wrote the first draft. All authors discussed the results and implications and developed the manuscript at all stages.

## Conflict of Interest

The authors declare that the research was conducted in the absence of any commercial or financial relationships that could be construed as a potential conflict of interest.
