# Supplementary material for: Sitravatinib, a Tyrosine Kinase Inhibitor, Inhibits the Transport Function of ABCG2 and Restores Sensitivity to Chemotherapy-Resistant Cancer Cells in vitro
Source: Front Oncol. 2020 May 12;10:700. doi: 10.3389/fonc.2020.00700 (PMC7236772; doi:10.3389/fonc.2020.00700)
Supplement: Supplementary file 1 [file Data_Sheet_1.docx]

**Table S1 Docking scores of the binding of thirty TKIs to human ABCG2 model (PDB code: 6ETI).**

| Drug name | Glide gscore^a^ (kcal/mol) |
| --- | --- |
| Sitravatinib | -13.248 |
| Merestinib | -11.486 |
| BMS-777607 | -11.461 |
| Gilteritinib | -10.269 |
| LOXO-101 | -8.531 |
| Belizatinib (TSR-011) | -8.294 |
| CA-4948 | -8.277 |
| G-749 | -8.201 |
| BLU-667 | -8.024 |
| ASP3026 | -7.986 |
| Sapitinib (AZD8931) | -7.871 |
| PCI 29732 | -7.714 |
| TAS6417 | -7.626 |
| S49076 | -7.500 |
| TP0427736 | -7.478 |
| AMG 925 | -7.463 |
| X-376 | -7.234 |
| KU14R | -6.853 |
| BMS-690514 | -6.727 |
| AG-1024 | -6.702 |
| TG101209 | -6.644 |
| QL47 | -6.547 |
| ENMD-2076 | -6.425 |
| Crizotinib (PF-02341066) | -6.287 |
| AZ1495 | -6.224 |
| Acalabrutinib | -5.975 |
| FF-10101 | -5.899 |
| Repotrectinib (TPX-0005) | -5.733 |
| UNC2250 | -5.132 |
| MK-5108 (VX-689) | -3.204 |

^a.^ Docking scores were obtained as described in the materials and methods section.

**Table S2. The effect of sitravatinib on the anticancer efficacy of chemotherapeutic drugs in drug-selected ABCG2-overexpressing cell lines.**

| Treatment | IC_50_ ± SD^a^ (μM) (RF^b^) | | | |
| --- | --- | --- | --- | --- |
|  | NCI-H460 | NCI-H460/MX20 | S1 | S1-M1-80 |
| **Mitoxantrone** | 0.0212 ± 0.0010 (1.00) | 2.6325 ± 0.0021 (124.44) | 0.0350 ± 0.0021 (1.00) | 5.4187 ± 0.2019 (154.93) |
| + Sitravatinib 0.75μM | 0.0207 ± 0.0013 (0.98) | 0.4319 ± 0.0038 (20.42) * | 0.0346 ± 0.0026 (0.99) | 0.6372 ± 0.0028 (18.22) * |
| + Sitravatinib 1.5μM | 0.0206 ± 0.0004 (0.97) | 0.2361 ± 0.0036 (11.16) * | 0.0361 ± 0.0011 (1.03) | 0.3058 ± 0.0126 (8.74) * |
| + Sitravatinib 3μM | 0.0210 ± 0.0006 (0.99) | 0.0648 ± 0.0049 (3.06) * | 0.0342 ± 0.0035 (0.98) | 0.1467 ± 0.0074 (4.20) * |
| + Ko143 3μM | 0.0204 ± 0.0005 (0.97) | 0.0605 ± 0.0001 (2.86) * | 0.0345 ± 0.0037 (0.99) | 0.1209 ± 0.0029 (3.46) * |
| **SN-38** | 0.0444 ± 0.0010 (1.00) | 6.5253 ± 0.0651 (147.01) | 0.0418 ± 0.0012 (1.00) | 4.4179 ± 0.1049 (105.63) |
| + Sitravatinib 0.75μM | 0.0475 ± 0.0009 (1.07) | 1.1517 ± 0.0420 (25.95) * | 0.0425 ± 0.0003 (1.02) | 0.3180 ± 0.0304 (7.60) * |
| + Sitravatinib 1.5μM | 0.0449 ± 0.0011 (1.01) | 0.4235 ± 0.0103 (9.54) * | 0.0438 ± 0.0013 (1.05) | 0.1823 ± 0.0263 (4.36) * |
| + Sitravatinib 3μM | 0.0468 ± 0.0007 (1.05) | 0.1352 ± 0.0018 (3.05) * | 0.0433 ± 0.0011 (1.04) | 0.0640 ± 0.0076 (1.53) * |
| + Ko143 3μM | 0.0429 ± 0.0010 (0.97) | 0.1217 ± 0.0051 (2.74) * | 0.0439 ± 0.0003 (1.05) | 0.0394 ± 0.0023 (0.94) * |
| **Topotecan** | 0.0508 ± 0.0006 (1.00) | 5.8046 ± 0.1420 (114.38) | 0.0532 ± 0.0035 (1.00) | 5.9900 ± 0.1838 (112.59) |
| + Sitravatinib 0.75μM | 0.0490 ± 0.0009 (0.97) | 1.0578 ± 0.0470 (20.84) * | 0.0522 ± 0.0006 (0.98) | 0.9235 ± 0.0163 (17.36) * |
| + Sitravatinib 1.5μM | 0.0518 ± 0.0034 (1.02) | 0.4389 ± 0.0350 (8.65) * | 0.0521 ± 0.0020 (0.98) | 0.6663 ± 0.0414 (12.52) * |
| + Sitravatinib 3μM | 0.0511 ± 0.0002 (1.00) | 0.2073 ± 0.0086 (4.08) * | 0.0577 ± 0.0030 (1.08) | 0.1696 ± 0.0031 (3.19) * |
| + Ko143 3μM | 0.0548 ± 0.0001 (1.08) | 0.2370 ± 0.0085 (4.67) * | 0.0579 ± 0.0068 (1.09) | 0.0633 ± 0.0088 (1.19) * |
| **Cisplatin** | 1.0357 ± 0.0279 (1.00) | 1.2057 ± 0.0270 (1.16) | 0.9079 ± 0.0506 (1.00) | 1.0167 ± 0.0472 (1.12) |
| + Sitravatinib 0.75μM | 1.0460 ± 0.0438 (1.01) | 1.1811 ± 0.0355 (1.14) | 0.9705 ± 0.0446 (1.07) | 1.0176 ± 0.0359 (1.12) |
| + Sitravatinib 1.5μM | 1.0705 ± 0.0318 (1.03) | 1.2040 ± 0.0210 (1.16) | 0.9575 ± 0.0526 (1.05) | 1.0170 ± 0.0141 (1.12) |
| + Sitravatinib 3μM | 1.0683 ± 0.0216 (1.03) | 1.1730 ± 0.0637 (1.13) | 1.0008 ± 0.0583 (1.10) | 1.0260 ± 0.0862 (1.13) |
| + Ko143 3μM | 1.0650 ± 0.0170 (1.03) | 1.1926 ± 0.0625 (1.15) | 0.9361 ± 0.0311 (1.03) | 1.0340 ± 0.0184 (1.14) |

* indicated that the IC_50_ values of chemotherapeutic drugs in drug-resistant cell line had significant statistical difference from the counterparts in corresponding sensitive cell line without inhibitor (*p* < 0.05).

^a.^ IC_50_ values were determined by modified MTT colorimetric assay as described in material and method section, and are shown as mean ± SD.

^b.^ Resistance fold (RF) were calculated by the IC_50_ values for chemotherapeutic drugs of drug-sensitive cells without inhibitor, divided by the IC_50_ values for chemotherapeutic drugs of drug-sensitive cells with inhibitor or drug-resistant cells in the absence or presence of inhibitor.

**Table S3. The effect of sitravatinib on the anticancer efficacy of chemotherapeutic drugs in gene-transfected ABCG2-overexpressing cell lines.**

| Treatment | IC50 ± SD^a^ (μM) (RF^b^) | | | |
| --- | --- | --- | --- | --- |
|  | HEK293/pcDNA3.1 | HEK293/ABCG2-482-R2 | HEK293/ABCG2-482-G2 | HEK293/ABCG2-482-T7 |
| **Mitoxantrone** | 0.0108 ± 0.0010 (1.00) | 0.1520 ± 0.0046 (14.10) | 0.4587 ± 0.0225 (42.55) | 0.2904 ± 0.0095 (26.94) |
| + Sitravatinib 0.75μM | 0.0107 ± 0.0007 (0.99) | 0.0418 ± 0.0044 (3.88) * | 0.0227 ± 0.0024 (2.11) * | 0.0415 ± 0.0065 (3.85) * |
| + Sitravatinib 1.5μM | 0.0110 ± 0.0004 (1.02) | 0.0317 ± 0.0019 (2.94) * | 0.0174 ± 0.0018 (1.61) * | 0.0251 ± 0.0055 (2.33) * |
| + Sitravatinib 3μM | 0.0115 ± 0.0001 (1.07) | 0.0200 ± 0.0025 (1.86) * | 0.0130 ± 0.0003 (1.21) * | 0.0183 ± 0.0008 (1.70) * |
| + Ko143 3μM | 0.0108 ± 0.0003 (1.00) | 0.0182 ± 0.0009 (1.69) * | 0.0125 ± 0.0010 (1.16) * | 0.0123 ± 0.0003 (1.14) * |
| **SN-38** | 0.0402 ± 0.0019 (1.00) | 0.7453 ± 0.0299 (18.52) | 0.9062 ± 0.0073 (22.52) | 0.8253 ± 0.0110 (20.51) |
| + Sitravatinib 0.75μM | 0.0484 ± 0.0001 (1.20) | 0.4519 ± 0.0143 (11.23) * | 0.3717 ± 0.0192 (9.24) * | 0.1663 ± 0.0897 (4.13) * |
| + Sitravatinib 1.5μM | 0.0435 ± 0.0017 (1.08) | 0.0845 ± 0.0031 (2.10) * | 0.0931 ± 0.0359 (2.31) * | 0.0855 ± 0.0142 (2.12) * |
| + Sitravatinib 3μM | 0.0468 ± 0.0049 (1.16) | 0.0457 ± 0.0040 (1.14) * | 0.0440 ± 0.0036 (1.09) * | 0.0457 ± 0.0008 (1.14) * |
| + Ko143 3μM | 0.0454 ± 0.0003 (1.13) | 0.0417 ± 0.0005 (1.04) * | 0.0433 ± 0.0007 (1.08) * | 0.0416 ± 0.0016 (1.03) * |
| **Topotecan** | 0.0735 ± 0.0041 (1.00) | 1.1793 ± 0.0999 (16.04) | 1.5760 ± 0.0394 (21.44) | 1.3335 ± 0.0708 (18.14) |
| + Sitravatinib 0.75μM | 0.0732 ± 0.0032 (1.00) | 0.6313 ± 0.0401 (8.59) * | 0.4624 ± 0.0049 (6.29) * | 0.1596 ± 0.0018 (2.17) * |
| + Sitravatinib 1.5μM | 0.0733 ± 0.0037 (0.99) | 0.2845 ± 0.0048 (3.87) * | 0.2358 ± 0.0178 (3.21) * | 0.1159 ± 0.0054 (1.58) * |
| + Sitravatinib 3μM | 0.0745 ± 0.0052 (1.01) | 0.1161 ± 0.0033 (1.58) * | 0.1237 ± 0.0161 (1.68) * | 0.0827 ± 0.0044 (1.12) * |
| + Ko143 3μM | 0.0740 ± 0.0044 (1.01) | 0.0968 ± 0.0028 (1.32) * | 0.1006 ± 0.0088 (1.37) * | 0.0850 ± 0.0094 (1.16) * |
| **Cisplatin** | 0.9965 ± 0.0304 (1.00) | 1.1165 ± 0.0502 (1.12) | 1.1259 ± 0.0199 (1.13) | 1.0830 ± 0.0255 (1.09) |
| + Sitravatinib 0.75μM | 0.9921 ± 0.0111 (0.99) | 1.1130 ± 0.0325 (1.12) | 1.1010 ± 0.0135 (1.10) | 1.0774 ± 0.0291 (1.08) |
| + Sitravatinib 1.5μM | 1.0505 ± 0.0856 (1.05) | 1.1430 ± 0.0028 (1.15) | 1.1309 ± 0.0030 (1.13) | 1.0980 ± 0.0594 (1.10) |
| + Sitravatinib 3μM | 1.0356 ± 0.0812 (1.04) | 1.1655 ± 0.0912 (1.17) | 1.1234 ± 0.0560 (1.13) | 1.1150 ± 0.0141 (1.12) |
| + Ko143 3μM | 0.9871 ± 0.0158 (0.99) | 1.1261 ± 0.0735 (1.13) | 1.1121 ± 0.0869 (1.12) | 1.1485 ± 0.0714 (1.15) |

* indicated that the IC_50_ values of chemotherapeutic drugs in drug-resistant cell line had significant statistical difference from the counterparts in corresponding sensitive cell line without inhibitor (*p* < 0.05).

^a.^ IC_50_ values were determined by modified MTT colorimetric assay as described in material and method section, and are shown as mean ± SD.

^b.^ Resistance fold (RF) were calculated by the IC_50_ values for chemotherapeutic drugs of drug-sensitive cells without inhibitor, divided by the IC_50_ values for chemotherapeutic drugs of drug-sensitive cells with inhibitor or drug-resistant cells in the absence or presence of inhibitor.
